# Acute macular neuroretinopathy secondary to central retinal artery occlusion

**DOI:** 10.1016/j.ajoc.2023.101793

**Published:** 2023-01-05

**Authors:** Emérentienne Sarrasin, Ariane Malclès, David Sarraf

**Affiliations:** a Department of Ophthalmology, Geneva University Hospitals, Geneva, Switzerland; b Faculty of Medicine, University of Geneva, Geneva, Switzerland; c Stein Eye Institute, University of California Los Angeles, Los Angeles, CA, USA

**Keywords:** CRAO, AMN, AMN secondary to CRAO, PAMM, ASHH, **AMN**, Acute Macular Neuroretinopathy, **APMPPE**, Acute Posterior Multifocal Placoid Pigment Epitheliopathy, **ASHH**, Angular Sign of HFL Hyperreflectivity, **CRAO**, Central Retinal Artery Occlusion, **CRVO**, Central Retinal Vein Occlusion, **DCP**, Deep Capillary Plexus, **EZ**, Ellipsoid Zone, **FA**, Fluorescein Angiogram, **HFL**, Henle Fiber Layer, **INL**, Inner Nuclear Layer, **IZ**, Interdigitation Zone, **OCT**, Optical Coherence Tomography, **OD**, Oculus Dexter, **ONL**, Outer Nuclear Layer, **OPL**, Outer Plexiform Layer, **OS**, Oculus Sinister, **PAMM**, Paracentral Acute Middle Maculopathy, **SD**, Spectral Domain

## Abstract

**Purpose:**

Acute Macular Neuroretinopathy (AMN) may be the result of deep retinal capillary plexus (DCP) impairment, but its mechanism remains elusive. A recent study has described simultaneous onset of Paracentral Acute Middle Maculopathy (PAMM) and AMN, suggesting a related pathogenic pathway. In this report, we analyze and describe the imaging characteristics of patients with concomitant Central Retinal Artery Occlusion (CRAO) and AMN and suggest a mechanistic pathway to explain this relationship.

**Observations:**

A total of 2 cases of CRAO, arteritic and non arteritic, were included in this report. At initial presentation, outer retinal layers were intact. At the two-week follow-up visit, both cases displayed Henle fiber layer hyperreflectivity and ellipsoid zone disruption consistent with AMN.

**Conclusions:**

Secondary development of AMN in CRAO is a new finding. DCP ischemia secondary to CRAO may lead to Henle fiber layer disruption, leading to the characteristic findings of AMN.

## 1 Introduction

Acute macular neuroretinopathy (AMN) is a rare macular disease typically affecting young women and causing one or more paracentral scotomas. On spectral domain-optical coherence tomography (SD OCT), the structural lesion features hyperreflectivity of the outer plexiform (OPL) and outer nuclear (ONL) layers, and focal disruption of the ellipsoid and interdigitation zone bands. Near-infrared reflectance can show a grey hyporeflective petaloid parafoveal lesion.^1^

Various triggers have been associated with AMN^2^ including non-specific flu-like illness or fever, oral contraceptive pill intake, exposure to epinephrine or ephedrine, trauma, or systemic hypotensive shock. The pathogenesis of AMN is still under debate. Some authors have implicated choroidal vascular flow defects,^3^ while others have identified deep capillary plexus (DCP) impairment,^4^ or both.^5^^,^^6^ A recent study described coincident paracentral acute middle maculopathy (PAMM) and AMN^7^ in 4 patients with Purtscher's retinopathy, 4 patients with central retinal vein occlusion (CRVO) and one patient with central retinal artery occlusion (CRAO), suggestive of a common mechanism involving the DCP.

In this report, we describe 2 cases of arteritic and nonarteritic CRAO, with sequential AMN onset after 2 weeks. This series provides insight into the pathophysiological mechanisms of AMN.

## 2 Case descriptions

### 2.1 Patient 1

**Fig. 1:**
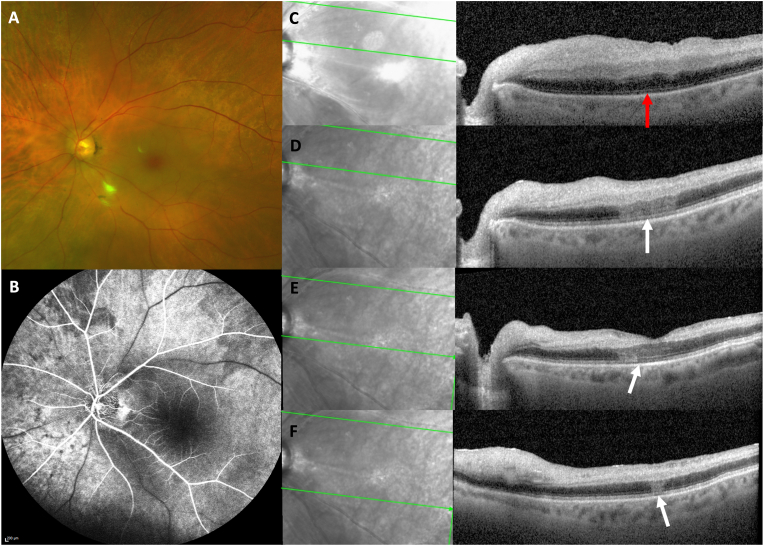
A. Baseline color fundus photography of patient 1 at symptom onset shows cherry red spot typical of a central retinal artery occlusion of the left eye B. Baseline fluorescein angiography of patient 1 at symptom onset shows physiological choroidal filling of the left eye C. Baseline Optical Coherence Tomography (OCT) of patient 1 at symptom onset shows hyperreflectivity and ischemia of the inner and middle retinal layers of the left eye. There is no visible alteration of the outer retinal layers (red arrow). D. Follow-up OCT of patient 1, two weeks after symptom onset, illustrating the same OCT section as in Fig. 1C. This figure shows hyperreflective lesion extending along the Henle fiber layer OS. Note the associated disruption of the ellipsoid and interdigitation zone bands (white arrow). E & F. Additional OCT B scans of patient 1 two weeks after onset show hyperreflective lesions inferior to the fovea radiating in the Henle fiber layer (white arrows). (For interpretation of the references to color in this figure legend, the reader is referred to the Web version of this article.)

An 86-year-old woman presented with acute vision loss in the left eye with visual acuity of light perception. Clinical examination revealed a cherry red spot and ischemic maculopathy (Fig. 1A). The OCT showed hyperreflectivity and ischemia of the inner and middle retinal layers (Fig. 1C). Fluorescein angiogram (FA) (Fig. 1B) did not show delayed choroidal filling but confirmed the diagnosis of CRAO OS. Temporal artery biopsy was negative for giant cell arteritis. CRAO was considered non arteritic. Two weeks later, the vision improved to hand motion OS and the macular OCT scan (Fig. 1D, E and F) revealed hyperreflective lesions of the outer retinal layers, radiating in the Henle fiber layer, superior and inferior to the fovea associated with ellipsoid zone (EZ) disruption consistent with AMN.

### 2.2 Patient 2

**Fig. 2:**
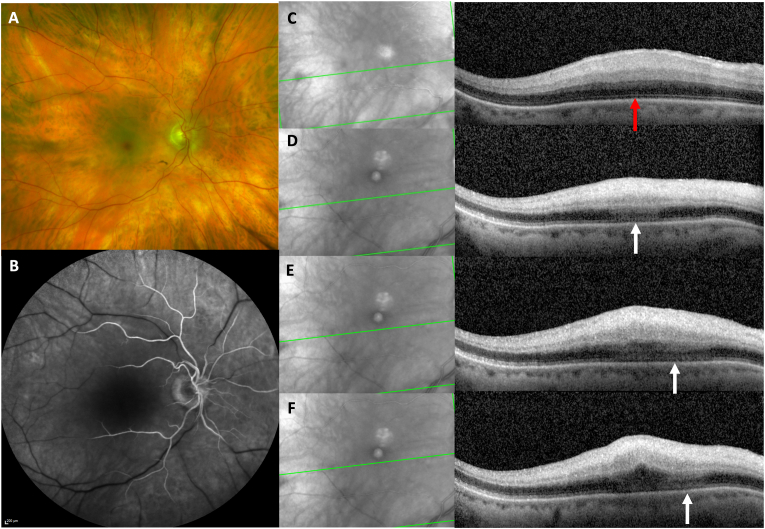
A. Baseline color fundus photography of patient 2 at symptom onset shows cherry red spot typical of a central retinal artery occlusion of the right eye B. Baseline fluorescein angiography of patient 2 at symptom onset shows physiological choroidal filling of the right eye C. Baseline OCT of patient 2 shows hyperreflectivity and ischemia of the inner and middle retinal layers of the right eye. There is no visible alteration of the outer retinal layers (red arrow). D. Two-week follow-up OCT scan (tracked to the OCT B scan shown in Fig. 2 C) of patient 2. This scan shows hyperreflectivity of the outer retinal layers with radiation along the Henle fiber layer and associated disruption of the ellipsoid zone (white arrow). E & F. Additional OCT sections from patient 2 two weeks after onset show hyperreflective lesions inferior to the fovea, radiating in the HFL (white arrows). (For interpretation of the references to color in this figure legend, the reader is referred to the Web version of this article.)

An 80-year-old man presented to the emergency room with acute vision loss in the right eye. He endorsed headache and jaw claudication for the past 3–4 days. Visual acuity in the right eye was light perception. Fundus examination OD showed a cherry red spot and ischemic maculopathy consistent with CRAO (Fig. 2A). OCT images displayed hyperreflectivity and ischemia of the inner and middle retinal layers (Fig. 2C) and choroidal filling time was preserved on the FA (Fig. 2B). Temporal artery biopsy confirmed the diagnosis of giant cell arteritis. The patient received high dose intravenous steroids. After 2 weeks, the visual acuity remained unchanged and OCT scans showed the development of an AMN lesion, inferior to the fovea. (Fig. 2 D - F).

## 3 Discussion

This paper describes 2 patients who presented with acute CRAO. Subsequent OCT 2 weeks later identified the development of AMN in each case. Iovino et al.^7^ described the association of CRAO with coincident PAMM and AMN but in this report we illustrate 2 additional cases that developed 2 weeks after the original baseline presentation. We believe AMN may be easily overlooked on OCT because ischemia and hyperreflectivity of the inner/middle retinal layers in CRAO may mask the details of the outer retinal layers thereby concealing underlying AMN. These OCT findings of AMN were not the result of transmission artifact as overlying hyperreflectivity of the inner/middle retinal layers was present which would be expected to cause hyporeflective shadowing. Moreover, associated EZ disruption was noted in each case.

Iovino et al.^7^ proposed that AMN may develop as a result of Henle fiber layer (HFL) disruption due to ischemia of the adjacent DCP. CRAO is associated with vascular impairment of the superficial and deep capillary plexuses. With ischemia of the DCP especially involving the distal collecting venules, adjacent HFL disruption may ensue. Ischemia of these unique elements may explain the development of AMN and the associated outer retinal and EZ disruption. Ramtohul et al.^8^ recently described the angular sign of HFL hyperreflectivity or ASHH with OCT that can be associated with AMN and other macular diseases such as laser induced maculopathy and acute posterior multifocal placoid pigment epitheliopathy (APMPPE). The 2- week delay as illustrated in the cases herein further supports the concept that the outer retinal abnormalities of AMN are secondary complications of inner and middle retinal ischemia.

## 4 Conclusions

In conclusion, we describe 2 patients with CRAO and subsequent development of AMN.

We believe ischemia of the middle retina layers likely causes disruption of the adjacent HFL leading to the characteristic outer retinal changes of AMN illustrated in the two cases presented in this report. Why certain regions within the macula show evidence of AMN while the outer layers in other areas remain intact requires further investigation.

## Patient consent

Consents to publish the case reports were not obtained. This report does not contain any personal information that could lead to the identification of the patient.

## Financial support

No funding or grant support

## Authorship

All authors attest that they meet the current ICMJE criteria for Authorship.

## Declaration of competing interest

The following authors have no financial disclosures: ES, AM.

DS: Consultant - 10.13039/100002429Amgen, 10.13039/100004326Bayer, Iveric Bio, 10.13039/100004336Novartis, and Optovue; research grants - 10.13039/100002429Amgen, Boehringer, 10.13039/100004328Genentech, Heidelberg, Optovue, 10.13039/100009857Regeneron, 10.13039/501100010383Topcon.
